# Exploratory Retrospective Study on Rehabilitation in Dogs Affected by Acute Idiopathic Polyradiculoneuritis

**DOI:** 10.3390/ani16193158

**Published:** 2026-10-08

**Authors:** Enrico Bruno, Elena Salvador, Federica Balducci, Michela Antonucci, Thomas Dalmonte, Giuseppe Spinella

**Affiliations:** 1 AniCura Ospedale Veterinario ‘I Portoni Rossi’, Via Roma, 57/A, 40069 Zola Predosa, BO, Italy; enrico.bruno@anicura.it (E.B.); federica.balducci@anicura.it (F.B.); antonucci.michelavet@gmail.com (M.A.); 2 Department of Veterinary Medical Sciences, University of Bologna, 40064 Ozzano dell’Emilia, BO, Italythomas.dalmonte2@unibo.it (T.D.)

**Keywords:** acute idiopathic polyradiculoneuritis, rehabilitation, dog

## Abstract

Acute Canine Idiopathic Polyradiculoneuritis (ACIP) is one of the most common acute-onset peripheral neuropathies in dogs. Since there is no specific therapy, rehabilitation represents the main supporting treatment for canine patients. This exploratory retrospective study analyzes the role and effectiveness of physiotherapy rehabilitation in the treatment of ACIP, evaluating the recovery times of spontaneous walking ability and potential correlation between the considered variables that could influence its progress. The study included 12 clinical cases affected by ACIP and submitted to a physiotherapy support plan for independent gait recovery. Patients were divided into two groups on the basis of the number of rehabilitation sessions required for spontaneous ambulation achievement: less than 40 (Group A) and more than 40 sessions (Group B). Statistical analysis showed no significant association between qualitative variables. Given the inherent methodological limitations and small cohort size, these preliminary findings should be interpreted with caution. However, the data tentatively suggest that intensive rehabilitation strategies might support a more rapid recovery of independent ambulation, even though the number of moderate and maintenance sessions did not differ significantly between the groups. Consequently, further studies with statistical power are required to confirm these trends.

## Simple Summary

Scientific evidence on Acute Canine Idiopathic Polyradiculoneuritis (ACIP) remains limited, and several aspects of the disease are still poorly understood. This retrospective study investigated a rehabilitation multimodal approach in dogs affected by ACIP, monitoring their return to independent ambulation. No significant association was observed for qualitative variables in dogs with a faster recovery compared to dogs with a slower return to independent ambulation. Despite the study’s limitations—often related to owner compliance—the results obtained from this small sample could suggest that a more intensive rehabilitation approach could lead to a faster return to independent ambulation. However, causality cannot be inferred because of the retrospective nature of this study and the potential differences in owner compliance. Further prospective studies with a wider sample are needed.

## 1. Introduction

The Coonhound Paralysis (CP) or Acute Canine Idiopathic Polyradiculoneuritis (ACIP) is the most common form of acute-onset polyneuropathy diagnosed in dogs and, more rarely, in other species like cat, goat and horse [1,2]. ACIP is a sporadic peripheral neuropathy and an acute inflammatory disease on a suspected immunomediated origin, that primarily involves the axons and myelin of the ventral spinal roots and, at the same time, the most peripheral component of the nerve [3,4]. Although the exact pathogenesis of ACIP remains to be fully elucidated, accumulating evidence suggests that it is mediated by an aberrant immune response resembling the pathogenic mechanisms underlying the Guillain–Barré syndrome (GBS) in humans [1]. Consequently, ACIP has been proposed as a spontaneous canine model of GBS [5]. Guillain–Barré syndrome is an acute immune-mediated inflammatory polyradiculoneuropathy encompassing both demyelinating and axonal variants, characterized by immune-mediated injury to the peripheral nervous system (PNS). Approximately 75% of GBS cases are preceded by an infectious illness or, less commonly, by immunization, suggesting that molecular mimicry and other infection-triggered immune mechanisms play a pivotal role in disease development [4,6].

ACIP affects ventral nerve roots of spinal nerves (especially those in the lumbosacral region) and the peripheral component of nerves, determining several levels of axonal degeneration, paranodal and segmental demyelination, as well as infiltration of inflammatory cells [1,7,8]—all changes that, taken together, explain the typical clinical signs.

Clinical signs have an acute onset, typically occurring 7–14 days after a triggering event. In approximately 80% of cases, weakness initially affects the pelvic limbs before ascending to involve the thoracic limbs, leading to ambulatory or non-ambulatory tetraparesis or, in severe cases, flaccid tetraplegia due to lower motor neuron dysfunction. The duration of the disease generally ranges from several weeks to months, with gradual recovery [1,4]. Neurological examination is characterized by generalized hyporeflexia or areflexia of the spinal reflexes, except for preservation of the perineal reflex, together with diffuse hypotonia or muscle atony. Marked neurogenic muscle atrophy develops rapidly, particularly in recumbent patients [1,9]. Cranial nerves may be involved (especially vagus and facial nerves) and, although proprioception and pain sensitivity are generally preserved, some animals may experience hyperesthesia. In severe cases, the disease can progress to the point of compromising breathing, requiring ventilator support [1,4,9,10,11,12].

According to Pellegrino et al. (2018), ACIP represents 6% of neuromuscular diseases and 0.35% of all neurological disorders in dogs, although the condition is probably underdiagnosed in clinical practice [4]. Diagnosis is primarily based on signaling, medical history and identification of clinical signs typical of rapidly progressive polyneuropathy through general clinical examination and neurological examination. It is essential to rule out other possible causes of peripheral neuropathy. For this reason, routine blood and urine tests, thyroid functional parameters assessment, serological tests for antibodies against *T. gondii*, *N. caninum* and acetylcholine receptors, as well as chest X-rays and abdominal ultrasounds, are usually performed to exclude metabolic, infectious or neoplastic causes. Since there is no specific test to confirm the disease, exams such as electromyography, cerebrospinal fluid examination, neuromuscular biopsy, ELISA tests and the search for “anti-GM” ganglioside antibodies can provide useful diagnostic support [1,13,14].

The prognosis is generally favorable, but the outcome depends on the severity of the disease, with some dogs that may not recover completely, keeping neurological deficits, and others that may develop complications, recurrences or, in severe cases, require euthanasia [1,9]. Usually, recovery times can range from 3 to 5 weeks in patients with mild to moderate forms, up to 3 [3] or 4–6 months in more severe cases [1].

Scientific evidence on ACIP remains limited, as well as studies capable of providing scientific evidence on various aspects of the disease. Over the last decade, research has primarily focused on identifying risk factors and pathogenic mechanisms, discovering disease-specific biomarkers, and improving diagnostic and therapeutic approaches, as well as advancing the understanding of prognosis and disease progression [3,11,12,14,15,16,17,18].

Despite the obvious usefulness of physical therapy (manual and instrumental) in supporting the functional recovery of dogs with ACIP, studies investigating its application are rare. Information available regarding intervention and recovery times, rehabilitation protocols and the effectiveness of different therapeutic techniques remain limited and there are no established guidelines. Most rehabilitation approaches are based on clinical experience and individual observations by attending physiotherapists rather than systematic and validated studies.

This retrospective clinical study aimed to investigate the role of rehabilitation in dogs affected by Acute Canine Idiopathic Polyradiculoneuritis, with particular attention to the time to recovery of independent ambulation and the variables potentially associated with this outcome.

## 2. Materials and Methods

This study is based on a retrospective review of the medical records of patients affected by ACIP and referred to the Veterinary Hospital “I Portoni Rossi” between 2017 and 2024. The study is part of a wide research project approved by the Animal Welfare Committee of Bologna University (Project protocol number: ID 4764; further authorization: n.22739/2026).

To select the patients, specific inclusion and exclusion criteria were developed.

Inclusion criteria included patients:

- Presented with ACIP as their initial differential diagnosis, based on the diagnostic process performed and clinical course. The process had to include a complete general clinical examination, a neurological examination performed by a specialized neurologist and a hematobiochemical profile;
- Referred at the center with acute, non-deambulatory flaccid tetraparesis as the principal sign;
- Submitted to a physiotherapy treatment immediately after the clinical diagnosis;
- Discharged ambulatory from the center.

Exclusion criteria included patients:

- Deambulatory at the beginning of the physiotherapy treatment;
- Discharged non-deambulatory upon request of the owner or transfer to another center;
- Euthanized due to the development of complications related to the disease.

The following data were collected for all included patients: breed; sex; age (months); body weight (kg); comorbidities; spontaneity or lack of physiological functions such as defecation and urination; manual and instrumental activities performed; diagnostic protocol applied; administration or lack of post-diagnosis antibiotic therapy; time (in days) taken between the onset of clinical symptoms and the start of physical therapy; interval (in days) between the onset of non-ambulatory flaccid tetraparesis and the start of physical therapy; total duration of physical therapy (in days) until spontaneous resumption of walking; total number of sessions until spontaneous resumption of walking; number of days of “moderate” physiotherapy (2 sessions per day) and “maintenance” physiotherapy (1 session per day).

With regard to “weight”, in order to detect a possible association between the size of the patients and the other variables taken into consideration, weight was categorized by dichotomizing the variable into “small size” (≤15 kg) and “medium-large size” (>15 kg).

All patients included in the study underwent also a neurological examination and a complete physiatrist evaluation, with particular attention to the following aspects: postural and proprioceptive reactions, gait analysis (with specific aids such as braces and harnesses), cranial nerve examination, spinal reflex assessment, analysis of muscle tone and tropism and measurement of joint ROM in decubitus.

The physical therapy protocol consisted of 2 sessions per day, one in the morning and one in the afternoon, from Monday to Friday. Rehabilitation procedures were adapted according to the patient’s clinical status and the owner’s circumstances. If admitted to a day-hospital, between the two sessions (morning and afternoon treatment), the dogs were kept in cages on a soft, anti-bedsore mattress and physiological needs (such as urination and defecations) were managed according to specific patients’ conditions.

The rehab plan included: (1) manual techniques, such as muscle massages, passive exercises (PROM, stretching, flexor reflex stimulation), active-assisted exercises and/or active exercises (at the beginning maintenance of the sternal position, followed by maintenance of the quadrupedal station using a physioroll or physioball, proprioceptive board and assisted walking on an underwater treadmill—UWTM); (2) modalities, such as laser therapy, tecar-therapy and electrostimulation, once per day.

Underwater treadmill therapy was usually introduced a few days after the start of rehabilitation to allow adequate clinical observation of the patient, assess its individual needs, and ensure that the acute phase of the disease had subsided. The starting water activity protocol included sessions lasting only one minute, with the speed adjusted according to the patients’ size and the water level set at the level of the femoral greater trochanter. Every day the difficulty of the exercise was gradually increased, raising both the time and the speed of the activity.

Laser therapy was daily performed using an ASA Laser Mphi Vet Orange device (AsaLaser, Arcugnano (VI), Italy) with pulsed sequential emission. The parameter set included a frequency of 1168 Hz, a wavelength of 808 nm and an energy density of approximately 4 J/cm^2^. The laser light was applied to specific muscle groups, including the pectoral muscles, triceps brachii, quadriceps femoris, biceps femoris and the pectineus.

The tecartherapy treatment was performed using the—DVET 900 device (White Medical & Beauty, Pieve di Cento (BO), Italy), which operates at a working frequency of 1 MHz. Electromagnetic energy was applied using a manual bipolar capacitive electrode, with an output voltage of approximately 320 V and a power of approximately 100 W. Areas of application were selected based on clinical needs, including: the spine (especially cervical and lumbar regions) to have an effect on the spinal roots involved and reduce the local inflammation; joints (such as knees, elbows, carpus and tarsus) to promote mobility and counteract the instability resulting from a lack of support from muscle mass; trigger points for treating muscle contractures or hypertonia caused by compensatory postures or prolonged immobility.

Neuromuscular electrical stimulation treatment (NMES) was performed using the GLOBUS STIM Vet 4000 device (GlobusVet, Codognè (TV). Italy) with a specific preset program for muscle strengthening and with a working frequency between 30 and 60 Hz. The application was adjusted to muscle mass of front and hind limbs depending on functional needs of the single patient.

### Statistical Analysis

For all included patients a descriptive analysis (mean ± standad deviation; median) was performed. Afterward, patients were retrospectively divided into 2 groups (Group A and Group B) depending on the number of physical therapy sessions needed to regain the physiological and spontaneous ability to walk: less than 40 sessions for the patients in Group A and more than 40 sessions for those in Group B. The specific threshold of 40 sessions employed for group allocation was established based on a combination of published data, initiated by a pilot study conducted by Asnal et al. in 2019 that observed a mean number of rehabilitation sessions for the recovery of 38 sessions in a group of 5 dogs affected by ACIP [19]. This preliminary data was combined with observations of the paper of Hirschvogel et al. in 2012, where dogs, affected by ACIP and treated with a rehabilitation protocol and supportive care, could show recovery in a period between 3 and 5 weeks, considering also the gravity of clinical symptoms [3]. Finally, the 40 sessions cut-off (about 4 weeks of rehabilitation) was also defined based on our standard rehabilitative protocol, consisting of 2 sessions per day, from Monday to Friday, for a total of 10 sessions per week and 40 sessions per month (i.e., 4 weeks, corresponding to the estimated minimum healing time). However, we underline that in the clinical practice, the protocol was adapted to the specific needs of both the patient and the owner (for example, for reasons of financial or organizational availability); this particular condition makes it impossible to rule out the potential for bias. Maintaining therapy or moderate therapy consisted of 1 session per day and 2 sessions per day, respectively. Consequently, this cut-off could be considered as an exploratory, pragmatic and center-specific categorization. Treatment frequency (moderate vs. maintenance therapy) was re-evaluated upon clinical improvement—i.e., recovery of independent ambulation or transition to ambulatory tetraparesis—and adjusted in agreement with the owner according to the patient’s clinical status and the owner’s compliance and circumstances. As this was a retrospective study, this decision process could not be standardized across the full cohort.

Continuous variables included: age (months), body weight (kg), duration of clinical signs (S-F), time from diagnosis/onset to the beginning of physiotherapy (P-F), duration of the moderate-intensity physiotherapy phase (F-RSD), and duration of the maintenance physiotherapy phase (S-RSD).

Categorical variables included: sex, body size category (small, ≤15 kg; big, >15 kg), presence of comorbidities, defecation, and empirical antimicrobial treatment pending infectious-disease test results. For continuous variables, the distribution of data and equality of variances was assessed separately within Groups A and B using the Shapiro–Wilk and Levene test, respectively. For variables meeting the normality and homoschedasticity assumption in both groups, two-sample Student’s *t*-test was applied, while when variables showed normality distribution and heteroschedasticity, two-sample Welch *t*-test was performed. Finally, when variables did not meet the normality assumption, the Mann–Whitney test was applied.

Furthermore, categorical variables were compared between Groups A and B using Fisher’s exact test. Particularly, for 2 × 2 contingency tables, odds ratios (ORs) and corresponding 95% confidence intervals (95% CIs) were calculated as measures of the strength and precision of the association between each categorical variable and group allocation. Moreover, to account for multiple comparisons among the categorical variables evaluated using Fisher’s exact tests, *p*-values were adjusted according to the Holm step-down procedure.

Statistical analyses were performed using R 4.5.0 (R foundation for statistical computing; Vienna, Austria; https://www.R-project.org/ accessed on 18 September 2026). A *p*-value < 0.05 was considered statistically significant.

## 3. Results

The retrospective review of the medical records allowed us to identify a total of 21 patients admitted to the rehabilitation center for ACIP diagnosis.

Of these 21 subjects, 12 were included in the study population because they satisfied the inclusion criteria and nine were excluded for the following reasons: eight dogs were discharged non-deambulatory upon request of the owner or transfer to another center and one dog was euthanized due to the development of aspiration pneumonia.

The study population consisted of dogs of different breeds: four mixed-breed dogs, two German Shepherds, two Labrador Retrievers, one Golden Retriever, one Central Asian Shepherd, one Shih-tzu and one English Setter. The average age of these patients was 85.42 ± 53.23 months (median 88.5 months) and the average weight was 28.72 ± 17.63 kg (median 27.4 kg). As for size, 10 dogs were classified as medium-large size (>15 kg) and two as medium-small (≤15 kg). As for gender and reproductive status, however, the group was composed of four intact males (IM), two neutered males (NM), four intact females (IF) and two spayed females (SF). All included dogs had a non-ambulatory flaccid tetraparesis.

Afterward, patients were divided into the two groups described above: Group A composed of six dogs who performed less than 40 physiotherapy sessions until spontaneous ambulatory ability and Group B composed of six patients who performed more than 40 physiotherapy sessions.

In Group A (six patients) there were, besides a mixed-breed dog, two German Shepherds, one Golden Retriever, one Labrador Retriever and one Central Asian Shepherd. Of these, one dog was IM, one was NM and four were IF. The average age was 68.33 ± 57.78 months (median 66.5 months) and the average weight was 36.97 ± 19.64 kg, (median 31.05 kg). The average time interval between the onset of clinical symptoms and the start of physical therapy was 6.67 ± 3.14 days (median 5.5 days). The average time interval between the onset of non-ambulatory flaccid tetraparesis and the start of physical therapy was 4.67 ± 1.86 days (median 5 days). The average duration of physical therapy (in days) until spontaneous resumption of walking was 14.33 ± 5.75 days (median 16 days) while the total number of sessions until spontaneous resumption of walking is 22.17 ± 13.04 sessions (median 23.5 sessions). On average, patients of Group A performed 16.33 ± 14.49 days of moderate therapy (two sessions per day) (median 13.5 days) and 5.67 ± 3.56 days of maintenance therapy (one session per day) (median 5.5 days). At the beginning of the rehabilitation program, five patients of this group were still taking Clindamycin (one of five received Clyndamycin and Doxociclin) as empirical antibiotic drug therapy. The antimicrobial therapy was suspended after serological results. In one of the patients, serology revealed an IgG titer for *N. caninum* equal to 1:40; meanwhile, all the other patients were negative to serology for *T. gondii* and *N. caninum*. No comorbidities were reported in the patients included in this group.

In Group B (six patients) there were, besides three mixed-breed dogs, breeds such as Shih-Tzu, English Setter and Labrador Retriever. Of these, three patients were IM, one was NM and two were SF. The average age was 102.50 ± 46.84 months (median 105.5 months) and the average weight was 20.47 ± 11.62 kg (median 18.75 kg). The average time interval between the onset of clinical symptoms and the start of physical therapy was 14.67 ± 15.81 days (median 7.5 days). The average time interval between the onset of non-ambulatory flaccid tetraparesis and the start of physical therapy was 5.83 ± 5.38 days (median 5 days). The average duration of physical therapy (in days) until spontaneous resumption of walking was 52.67 ± 20.79 days (median 51days), while the total number of sessions until spontaneous resumption of walking was 88.5 ± 33.58 sessions (median 75 sessions). On average, patients of Group B performed 57.83 ± 59.55 days of moderate therapy (median 44 days) and 22.17 ± 36.99 days of maintenance therapy (median 6.5 days). At the beginning of the rehabilitation program, two patients of this group were still taking Doxycycline or Clindamycin as antibiotic drug therapy. In three of the patients, serology revealed an IgM titer for *T. gondii* equal to 1:20, 1:40 and 1:50, respectively; meanwhile, all the other patients were negative to serology for *T. gondii* and *N. caninum*. Moreover, two dogs presented a comorbidity: one case had a cortical adenoma and one case had a stage B1 heart disease.

Data are summarized in Table 1 and Table 2.

Based on the division of patients into the two groups, Table 2 shows that two quantitative variables resulted statistically significant (indicated with an asterisk): average duration of physical therapy (in days) until spontaneous resumption of walking and total number of sessions until spontaneous resumption of walking, respectively.

Categorical variables, submitted to Fisher’s exact test, showed no statistically significant association.

Consequently, no statistically significant association was observed between sex and group allocation (Fisher’s exact test, *p* = 0.567; Holm-adjusted *p* = 1.000). Particularly, females were more frequent in Group A (4/6, 66.7%) than in Group B (2/6, 33.3%), whereas males showed the opposite distribution. The estimated odds ratio (OR) was approximately 3.53, indicating higher odds of females relative to males in Group A compared with Group B. However, the 95% confidence interval was wide and included the null value of 1, indicating substantial uncertainty around the effect estimate. Thus, although a numerical imbalance in sex distribution was observed between groups, this difference was not statistically significant.

Moreover, no statistically significant association was observed between body size category and group allocation (Fisher’s exact test, *p* = 0.455; Holm-adjusted *p* = 1.000). All subjects in Group A were classified as big (6/6, 100%), whereas in Group B, 4/6 subjects (66.7%) were classified as big and 2/6 (33.3%) as small. The estimated OR was infinite because no small subjects were present in Group A, resulting in a zero cell in the contingency table. Accordingly, the 95% confidence interval was very wide (95% CI: 0.194 to infinite) and included the null value of 1. Therefore, although body size showed a numerical imbalance between groups, no statistically significant association was detected, and the effect estimate was highly uncertain because of the small sample size and the presence of a zero cell.

Similarly, no statistically significant association was observed between the presence of comorbidities and group allocation (Fisher’s exact test, *p* = 0.455; Holm-adjusted *p* = 1.000). All subjects in Group A had no comorbidities (6/6, 100%), whereas in Group B, 4/6 subjects (66.7%) had no comorbidities and 2/6 (33.3%) had at least one comorbidity. The estimated OR was infinite because no subjects with comorbidities were present in Group A, resulting in a zero cell in the contingency table. The corresponding 95% confidence interval was therefore very wide (95% CI: 0.194 to infinite) and included the null value of 1. Although a numerical imbalance in comorbidity status was observed between groups, no statistically significant association was detected, and the effect estimate remained highly uncertain because of the limited sample size and the zero cell.

Furthermore, no statistically significant association was observed between empirical antimicrobial treatments and group allocation (Fisher’s exact test, *p* = 0.5455). Empirical antimicrobial treatment was reported in 5/6 subjects (83.3%) in Group A and in 3/6 subjects (50.0%) in Group B. The estimated odds ratio was 5 (95% CI: 0.34–72.77). Given the coding and ordering of the contingency table, this odds ratio refers to subjects not receiving empirical antimicrobial treatment relative to those receiving it; thus, values below 1 indicate that absence of empirical antimicrobial treatment was relatively less frequent in Group A than in Group B. However, the 95% confidence interval was very wide and included the null value of 1, indicating substantial uncertainty in the effect estimate. Therefore, although empirical antimicrobial treatment was numerically more frequent in Group A, the association with group allocation was not statistically significant.

Finally, no statistically significant association was observed between defecation and group allocation (Fisher’s exact test, *p* = 1.000; Holm-adjusted *p* = 1.000). Defecation was reported in 5/6 subjects (83.3%) in both Group A and Group B, while absence of defecation was observed in 1/6 subjects (16.7%) in each group. The estimated OR was 1.00, indicating identical observed odds between the two groups. The 95% confidence interval was wide and included the null value of 1, reflecting the considerable uncertainty associated with the small sample size. Therefore, no evidence of an association between defecation and group allocation was observed.

It is noteworthy that after adjustment for multiple comparisons using the Holm procedure across the five Fisher’s exact tests, all adjusted *p*-values were equal to 1.000, and none of the categorical variables showed a statistically significant association with group allocation.

## 4. Discussion

The aim of this study was the analysis and the evaluation of the role of physiotherapy in treatment of dogs affected by ACIP, with a focus on timing of spontaneous ambulatory ability and on potential factors that could influence the recovery. Results showed no significant association between the qualitative variables. For quantitative variables, while the number of moderate and maintenance sessions did not statistically differ between the groups, the significant difference in the time to independent ambulation merely hints at the possibility that shorter intervals between sessions might support a faster recovery of autonomous walking. However, given the study’s limitations (principally concerning the retrospective nature and the owner compliance), these outcomes should be interpreted cautiously.

Our study population is partially in line with others of previous publications with regard to the following variables: breed, age and sex [1,3,12,16,17] and no significant differences were reported between the two groups.

However, the variable “Sex” could play a role in the occurrence of ACIP, but further studies are necessary to evaluate this relatedness. In support of this hypothesis, it is known that, in humans, GBS is more frequent in males than in females [6,20]. So, if sex influences GBS incidence, it is plausible to hypothesize that it could have a role also in ACIP.

Despite the widely recognized clinical importance of a multimodal rehabilitation approach in patients affected by ACIP [1,10,13,19], scientific studies that explore its application are still scarce. The limited veterinary literature on ACIP reflects the broader challenges associated with the diagnosis and management of peripheral neuropathies. As emphasized by Cuddon (2002), these disorders remain among the most difficult conditions to diagnose and treat in veterinary neurology [1]. Consequently, evidence-based rehabilitation protocols for ACIP are lacking. However, given the strong clinical and pathophysiological similarities between ACIP and Guillain–Barré syndrome (GBS), findings from the human medical literature may offer a valuable framework for guiding rehabilitation in affected dogs.

For both conditions, the primary objectives of physiotherapy encompass minimizing acute-phase complications, preserving and enhancing muscle mass to prevent contractures, and delaying or mitigating neurogenic muscular atrophy. Additionally, interventions aim to maintain joint mobility and optimal range of motion (ROM), prevent secondary complications, restore lost motor function, and attenuate long-term residual neurological deficits [1,21,22].

Both in humans and dogs, the therapeutic approach is multimodal. Studies in the literature show that multimodal rehabilitation, meant as a combination of therapeutic, manual and instrumental interventions, seems to have a synergistic effect in restoring functionality. In general, human patients not submitted to physiotherapy during hospitalization had a lower quality recovery than those who did it [23,24].

A generic rehabilitative program for GBS combines manual therapies, physical exercises, relaxation techniques and instrumental therapies aimed to promote the functional recovery. Nervous transcutaneous electrostimulation (TENS) is the most commonly used, especially for pain management. The program is defined “generic” because multidisciplinary rehabilitation is always adjusted to the specific needs of each patient, varying both in content and intensity of treatment, based on the severity of neurological impairment and the rehabilitation goals set [24,25].

In the study of Khan et al. in 2011, performed on GBS patients, it is shown that, in post-acute/chronic forms of disease, a high-intensity rehabilitation program is significantly more effective than a low-intensity one [25].

Although a standardized rehabilitation protocol served as the basis for treatment, the therapeutic plan was individualized according to each patient’s clinical status and the owner’s circumstances. Consequently, the intensity of rehabilitation varied among patients, with some dogs undergoing a more intensive rehabilitation regimen than others: maintaining therapy (one session per day) or moderate therapy (two sessions per day).

This result suggests that physiotherapy can play a crucial role in promoting faster recovery, also without strictly standardized protocols. Moreover, it is proven by human medicine that an early treatment is essential to achieve a better prognosis [23,26]. However, further prospective studies are required to confirm whether this rehabilitation protocol provides superior outcomes, particularly in patients with prolonged recovery times.

Similarly, to ACIP, to improve mobility and prevent joint stiffness, GBS treatment includes passive mobility exercises (PROM and stretching), active-assisted exercises and active resistance exercises that involve the use of external resistance to strengthen the muscles.

With regard to stretching in humans, specific proprioceptive neuromuscular facilitation techniques are used to restore joint ROM and improve flexibility and muscle strength. These techniques include the “contraction–relaxation” method, the “antagonist muscle contraction” method, and a combination of the two called “contraction, relaxation and antagonist muscle contraction” [21,22].

Another key point of rehabilitation concerns the maintenance of posture and the prevention of orthopedic complications. Therefore, in both species, the frequent change in position is essential to prevent the risk of pressure sores and other complications related to prolonged immobility [21,22].

In patients suffering from ACIP, motor rehabilitation develops through a gradual process. Rehabilitation typically begins with exercises performed in recumbency to maintain passive joint range of motion and minimize muscle atrophy. As neurological function improves, dogs are progressively assisted with supportive devices, such as harnesses, slings, or wheelchairs, to facilitate weight-bearing and ambulation until independent walking is achieved. When the patient’s clinical condition permits, the use of an underwater treadmill (UWTM) represents an effective strategy for promoting gait recovery, as buoyancy reduces weight-bearing on the limbs while facilitating a more physiological gait pattern. Similarly, swimming is widely used as an adjunctive rehabilitation modality, allowing limb movements to be performed with minimal mechanical loading while improving muscle strength, endurance, and cardiovascular fitness [27,28,29,30]. To improve coordination, proprioception and balance, aids such as physioballs/physiorolls and proprioceptive boards are used [31].

The adoption of a multimodal approach leads to more effective recovery and improved quality of life in both species [20,21,32]. Indeed, multimodal approach is actually applied and adapted to a variety of pathologies, regardless of their nature, and represents a common basis for dealing with motor and neurological deficits, improving patients’ quality of life, promoting and accelerating functional recovery [33,34,35,36,37,38]—although a multimodal protocol precludes identifying which techniques offer a better outcome than others.

A key process for neurological recovery is neuroplasticity defined by Frank et al. (2018) as the ability of nervous tissue to undergo structural changes throughout life, both at the cellular and neural networks levels, resulting in functional changes [23].

It was demonstrated that physical exercise (passive, active-assisted and active exercises), especially when early started, is a safe and efficient way to increase BDNF (brain-derived neurotrophic factor) and NT-3 (neurotrophic factor-3) levels in spinal cord and skeletal muscle, offering functional benefits in terms of neuroplasticity, reduction in allodynia and motor recovery, with amplified and more significant results when integrated with other techniques such as NMES, laser therapy or acupuncture [23,39].

NMES can improve muscle performance and encourage recovery after neurological lesions; helps reduce disuse atrophy; stimulates motor control; and reduces spasticity thanks to the ability to activate larger motor units than those recruited by voluntary contractions. Although there is little evidence demonstrating an increase in voluntary strength with the exclusive use of NMES, combining it with active exercises offers the best functional results [23].

Laser therapy effectiveness, instead, is supported by various studies (especially connected to disk herniation) [40,41].

Although our findings showed the occurrence of comorbidities in rehabilitation patients with ACIP, Fisher’s exact test showed no significant association between the groups. Czerwik et al. in their case report describe a Jack Russel Terrier, male, 12 years, affected by ACIP and by myxomatous mitral valve degeneration at ACVIM B2 stage [14]. There is no scientifically proven correlation between heart disease and ACIP, but the interaction between the two pathologies can be encountered in clinical contexts where comorbidities are more frequent, such as in older dogs. In support of this, our results show that dogs suffering from both heart disease and ACIP fall within the adult/elderly age group. The presence of a heart disease, regardless of its correlation with ACIP, could still negatively affect the dog’s capacity to deal with symptoms and rehabilitation. For this reason, it is interesting to undertake further studies to explore more deeply the possible correlation between these two diseases with a wider sample.

The variable related to post-diagnosis empirical antimicrobial therapy resulted in no association between the dogs submitted to Groups A and B.

The use of antibiotics in ACIP treatment is not documented in the current literature and drugs such as Clindamycin and Doxycycline were used in some of the patients included in this study as empirical antimicrobial treatment, awaiting serology results. It should be noted that the majority of patients who received this therapy belonged to the early years (2017–2018) of our study period.

This approach could be justified by the possible infectious etiology of ACIP, hypothesized and investigated by some authors who highlighted a close association between T. gondii and ACIP or between Campylobacter upsaliensis, Campylobacter jejuni, and ACIP [12,16].

In a case report described by Kim et al. (2021), a mixed-breed bitch of 9 years, affected by ACIP and diagnosed with Clostridium perfringens and Campylobacter jejuni infection, was treated with support therapies which included also an antibiotic treatment, although the specific type of medication is not reported [42]. In our study, of the eight subjects treated with post-diagnosis antibiotic therapy, one patient showed IgG titers of 1:40 for *N. caninum* and one patient reported IgM titers 1:50 for *T. gondii*. These results could suggest a possible recent infection, although further tests are needed to confirm this hypothesis. According to Lorenz et al. (2012), in cases of confirmed protozoal polyradiculoneuritis, caused by *T. gondii* or *N. caninum*, the treatment should include Trimethoprim-Sulfadiazine (15 mg/kg OS, every 12 h) or Pyrimethamine (1 mg/kg OS, every 24 h) or Clindamycin (15–20 mg/kg OS, every 12 h), for a period of 4–6 weeks [2].

However, according to studies describing this approach in human clinical cases of GBS, antibiotic therapy is typically not indicated because the triggering infection is usually no longer present at the time of diagnosis. GBS is a post-infective autoimmune complication, but the infection itself solves before the onset of the disease [6] and what persists is the immune response and the resulting inflammation, which causes nerve damage. A similar mechanism could be the root cause of ACIP, suggesting that also in this affection the beginning infection is still overcome by the time symptoms appear.

In general, although our data suggests a positive therapeutic effect of physiotherapy on regaining ambulatory ability, the study presents some limitations related to various technical aspects. Among this: (1) the small sample of patients included in the population under statistical investigation can impact the strength of the results; (2) the lack of a control group that had not received any physical therapy post-diagnosis of ACIP, thus limiting the possibility of comparing the results obtained with those of a reference group; (3) the retrospective nature of the study and the absence of randomization, which could have helped reduce bias and increase the validity of the results; (4) the lack of follow-up of non-ambulatory discharged patients, that which resulted in the exclusion of these subjects from the final analysis, limiting the overall number of data; (5) the variability related to financial resources of the owners and their compliance; (6) most excluded dogs were removed because they were discharged non-ambulatory or transferred elsewhere, which may not be a random subset—these could plausibly be the more severely affected cases. This raises the possibility that the analyzed cohort is skewed toward more favorable outcomes; and (7) the lack of potentially useful information, such as details about the patients’ diet, which could affect the pathology and its possible infectious nature, and which could have contributed to understanding the factors that influence the incidence of the disease.

## 5. Conclusions

In conclusion, our results suggest that a more intensive physical therapy combined with a multimodal rehabilitation protocol can optimize the recovery of autonomous gait in dogs affected by ACIP. In this small retrospective cohort, more intesive rehabilitation sessions could be associated with membership in the faster recovery group;; however, causality cannot be inferred because treatment intensity was not standardized and may have been influenced by owner-related factors and clinical decision. Nonetheless, future studies incorporating larger patient cohorts are required to confirm these preliminary findings.

## Figures and Tables

**Table 1:** Data related to each single case included in the study. Gr = Group. Age: (months) at onset. Rec = Number recurrence after Reab. PD = days until the dog was able to walk unaided. PS = number of sessions until the dog was able to walk unaided. Comorb = Comorbidity. Symptoms days—Time interval between the onset of symptoms and the start of physical therapy. Start physio days—Time interval between the onset of non-deambulatory flaccid tetraparesis and the start of physical therapy. EAT= empirical antimicrobial treatment. GS = German shepherd; GR = Golden retriever; LR = Labrador retriever; CAS = Central Asian Shepherd; ES = English setter; IM = intact males; NM = neutered males; IF = intact females; SF = spayed females. Cl.15 = Clindamycin 15 mg/kg BID per os; Cl.12 = Clindamycin 12.5 mg/kg BID per os; DOX = Doxycycline 10 mg/kg SID per os. Neg = negative. N = none.

Gr	Breed	Age	Sex	Weight (kg)	Rec	PD	PS	Comorb	Symptoms Days	Start Physio Days	Moderate Physio Days	Maintenance Physio Days	EAT	Serology
A	GS	7	IF	20	0	20	36	N	6	5	18	7	N	Neg
	GR	33	IF	38	0	6	8	N	5	3	2	4	Cl.15	Neg
	LR	100	IF	26.7	0	9	18	N	3	2	9	3	Cl.12	Neg
	GS	104	IF	28.1	0	19	35	N	10	7	21	9	Cl.12 + DOX	Neg
	Mix-breed	150	NM	34	0	18	29	N	11	6	42	10	Cl.12	IgG 1:40 *N. caninum*
	CAS	16	IM	75	0	14	7	N	5	5	6	1	Cl.12	Neg
B	Shih-Tzu	44	SF	7.8	0	41	73	N	4	1	44	7	Cl.12	Neg
	Mix-breed	134	SF	19.5	0	61	118	Cortical adenoma	7	7	48	22	N	IgM 1:20 *T. gondii*
	Mix-breed	141	NM	29	0	34	69	N	19	15	39	0	Cl.12 + DOX	IgM 1:50 *T. gondii*
	ES	63	IM	18	2	74	141	ACVIM B1	5	3	174	6	Cl.12 + DOX	Neg
	LR	77	IM	38.5	0	29	53	N	8	8	42	2	N	IgM 1:40 *T. gondii*
	Mix-breed	156	IM	10	0	77	77	N	45	1	0	96	N	Neg

**Table 2:** Schematization of mean ± standard deviation (median) of values relating to the variables investigated in Groups A and B. S-F = average time interval between the onset of clinical symptoms and the start of physical therapy; P-F = average time interval between the onset of non-ambulatory flaccid tetraparesis and the start of physical therapy; F-RSD = average duration of physical therapy (in days) until spontaneous resumption of walking; S-RSD = total number of sessions until spontaneous resumption of walking; TMo = days of moderate therapy; TMa = days of maintenance therapy; *p*-value refers to *t*-test for independent samples for Age (months), P-F (days) and S-RSD (number), Welch *t*-test for F-RSD (days) and Mann–Whitney for Weight (kg). S-F (days), TMo (days) and TMa (days). * = statistically significant difference.

	Group A	Group B	*p*-Value
Age (months)	68.33 ± 57.78 (66.5)	102.50 ± 46.84 (105.5)	0.2868
Weight (kg)	36.97 ± 19.64 (31.05)	20.47 ± 11.62 (18.75)	0.1282
S-F (days)	6.67 ± 3.14 (5.5)	14.67 ± 15.81 (7.5)	0.4680
P-F (days)	4.67 ± 1.86 (5)	5.83 ± 5.38 (5.00)	0.6267
F-RSD (days)	14.33 ± 5.75 (16)	52.67 ± 20.79 (51.0)	0.0053 *
S-RSD (number)	22.17 ± 13.04 (23.5)	88.50 ± 33.58 (83.5)	0.0011 *
TMo (days)	16.33 ± 14.49 (13.5)	57.83 ± 59.55 (43.0)	0.1087
TMa (days)	5.67 ± 3.56 (5.5)	22.17 ± 36.99 (6.50)	0.2200

## Data Availability

The original contributions presented in this study are included in the article. Further inquiries can be directed to the corresponding author.
